# Unusual Cause of Mechanical Ileus: Abdominal Cocoon Syndrome

**DOI:** 10.5334/jbr-btr.1062

**Published:** 2016-02-17

**Authors:** Laurens J. Ceulemans, Nathalie P. Deferm, Sébastien Deferm, Robin A. V. Willaert, Julie T. Deferm, Filip M. Vanhoenacker

**Affiliations:** 1 Department of Abdominal Surgery, General Hospital Sint-Maarten, Duffel-Mechelen, Rooienberg 25, BE-2570 Duffel, Belgium; 2 Department of General Surgery, University Hospital Leuven, Campus Gasthuisberg, Herestraat 49, BE-3000 Leuven, Belgium; 3 Department of Radiology, General Hospital Sint-Maarten, Duffel-Mechelen, Rooienberg 25, BE-2570 Duffel, Belgium; 4 Department of Radiology, University Hospital Antwerpen, Wilrijkstraat 10, BE-2650 Edegem, Belgium, and Faculty of Medicine and Health Sciences, University of Ghent, Belgium

A 38-year-old black male patient was admitted with diarrhea and nausea over two days and aggravating pain in the meso- and epigastium that resolved after urination. He had no surgical history and only an episode of pulmonary tuberculosis five years earlier, for which he was properly treated. Physical examination revealed a tender and distended abdomen with clangorous sounds. His temperature was 36.1°C. Routine laboratory blood analyses were normal. An abdominal ultrasound revealed diffuse distention of the small intestine. A computed tomography (CT) scan showed a conglomerate of dilated small bowel loops in the meso- and hypogastrium, suggestive for a supravesical mechanical small bowel obstruction. Peritoneal thickening was seen in the right epigastrium (Figure A, white arrow). An explorative laparoscopy revealed a whitish, thickened membrane encapsulating the small bowels as a ‘cocoon’ (Figure B). Extensive adhesiolysis released an intestinal kinking in the lower abdomen, just above the bladder. No resection was needed. Histopathology of the membrane showed fibrocollagenous tissue with mixed inflammatory infiltrate.

**Figure A and B:**
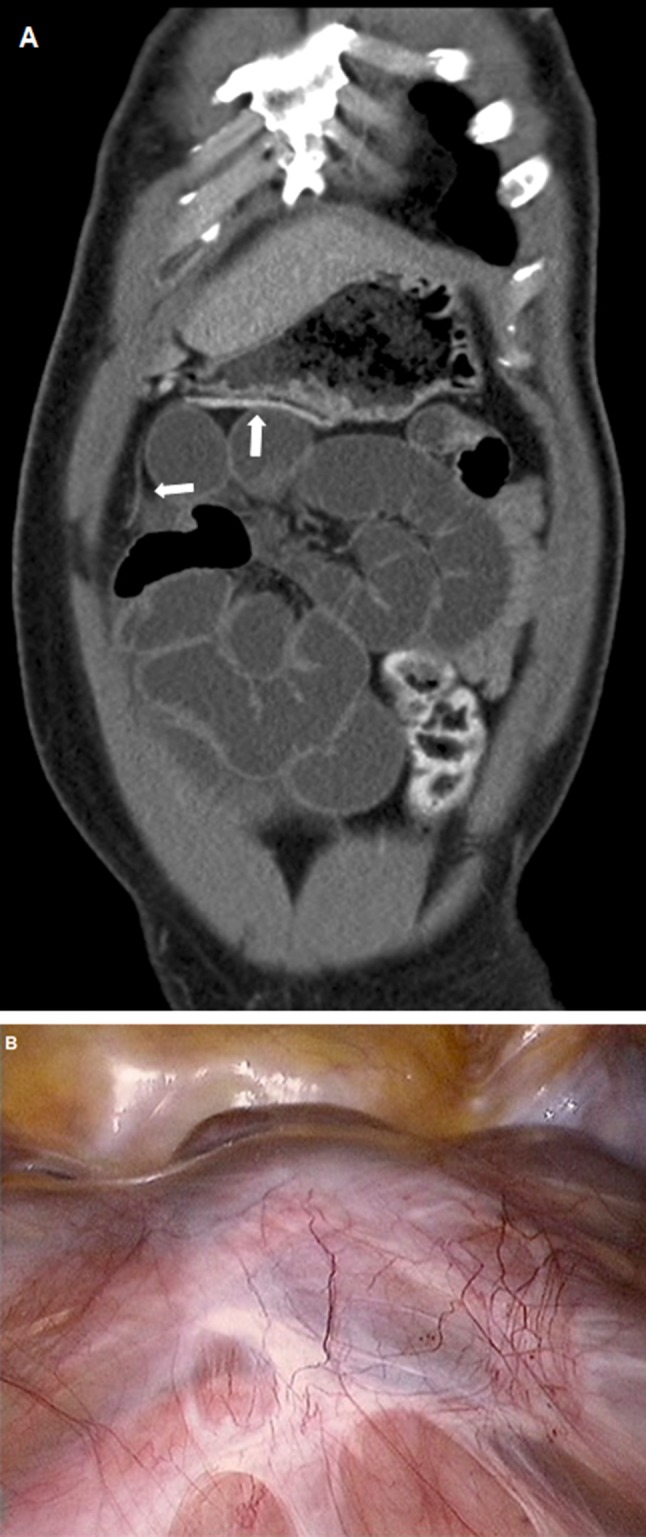


## Comment

Abdominal cocoon, or sclerosing encapsulating peritonitis, is a rare cause of intestinal obstruction which usually remains undiagnosed pre-operatively. The condition is divided in a primary or idiopathic form and a secondary form which is mostly seen after chronic ambulatory peritoneal dialysis, infection, or auto-immune disease that provoked a peritoneal inflammation. The condition is clinically characterized by an acute or sub-acute intestinal obstruction. Despite the fact that CT is mostly performed pre-operatively, the condition is often overlooked. Retrospective analysis of the images reveals the typical finding of abdominal cocoon as a concentration of the whole small bowel to the center of the abdomen encased by a soft tissue-density mantle, mimicking a cocoon. This coiling of the small bowel in a concertina-like fashion has been described as a “cauliflower-sign”. Other CT features include signs of obstruction, agglutination and fixation of intestinal loops, mural thickening, fluid collections, peritoneal thickening and enhancement [1].

In most of the cases, adhesiolysis is indicated. Perioperatively, a well-formed membrane is found covering or encasing the small intestine. Although the disease primarily involves the small bowel, it can involve other organs, such as the large intestine, liver, and stomach. Adhesiolysis and stripping the membrane of the intestine is usually easy and effective, hereby resolving the kinking and mechanical obstruction.

In the case described herein, we hypothesized that the abdominal cocoon-related obstruction was a late symptom of an earlier episode of tuberculosis with intra-abdominal manifestations. This was confirmed histologically since the primary or idiopathic form is mostly found to consist of a thin mesothelial layer, whereas the secondary form is described to be a thick fibrous structure that includes inflammatory cells – as found in our case – due to a previous episode of inflammation.

## Competing Interests

The authors declare that they have no competing interests.
